# Microbiota-associated risk factors for asymptomatic gut colonisation with multi-drug-resistant organisms in a Dutch nursing home

**DOI:** 10.1186/s13073-021-00869-z

**Published:** 2021-04-07

**Authors:** Quinten R. Ducarmon, Elisabeth M. Terveer, Sam Nooij, Michelle N. Bloem, Karuna E. W. Vendrik, Monique A. A. Caljouw, Ingrid M. J. G. Sanders, Sofie M. van Dorp, Man C. Wong, Romy D. Zwittink, Ed J. Kuijper

**Affiliations:** 1 Department of Medical Microbiology, Leiden University Medical Center, Leiden, The Netherlands; 2 Center for Microbiome Analyses and Therapeutics, Leiden University Medical Center, Leiden, The Netherlands; 3 Center for Infectious Disease Control, National Institute for Public Health and the Environment, Bilthoven, The Netherlands; 4 Department of Public Health and Primary Care, Leiden University Medical Center, Leiden, The Netherlands; 5 Department of Internal Medicine and Geriatrics, Onze Lieve Vrouwe Gasthuis (OLVG Hospital), Amsterdam, The Netherlands

**Keywords:** Gut microbiota, Multidrug-resistant organisms, Asymptomatic colonisation, Colonisation resistance, *Bifidobacterium*, Nursing home, Extended-spectrum beta-lactamase-producing *Enterobacterales*

## Abstract

**Background:**

Nursing home residents have increased rates of intestinal colonisation with multidrug-resistant organisms (MDROs). We assessed the colonisation and spread of MDROs among this population, determined clinical risk factors for MDRO colonisation and investigated the role of the gut microbiota in providing colonisation resistance against MDROs.

**Methods:**

We conducted a prospective cohort study in a Dutch nursing home. Demographical, epidemiological and clinical data were collected at four time points with 2-month intervals (October 2016–April 2017). To obtain longitudinal data, faecal samples from residents were collected for at least two time points. Ultimately, twenty-seven residents were included in the study and 93 faecal samples were analysed, of which 27 (29.0%) were MDRO-positive. Twelve residents (44.4%) were colonised with an MDRO at at least one time point throughout the 6-month study.

**Results:**

Univariable generalised estimating equation logistic regression indicated that antibiotic use in the previous 2 months and hospital admittance in the previous year were associated with MDRO colonisation. Characterisation of MDRO isolates through whole-genome sequencing revealed *Escherichia coli* sequence type (ST)131 to be the most prevalent MDRO and ward-specific clusters of *E. coli* ST131 were identified. Microbiota analysis by 16S rRNA gene amplicon sequencing revealed no differences in alpha or beta diversity between MDRO-positive and negative samples, nor between residents who were ever or never colonised. Three bacterial taxa (*Dorea, Atopobiaceae* and *Lachnospiraceae* ND3007 group) were more abundant in residents never colonised with an MDRO throughout the 6-month study. An unexpectedly high abundance of *Bifidobacterium* was observed in several residents. Further investigation of a subset of samples with metagenomics showed that various *Bifidobacterium* species were highly abundant, of which *B. longum* strains remained identical within residents over time, but were different between residents.

**Conclusions:**

Our study provides new evidence for the role of the gut microbiota in colonisation resistance against MDROs in the elderly living in a nursing home setting. *Dorea*, *Atopobiaceae* and *Lachnospiraceae* ND3007 group may be associated with protection against MDRO colonisation. Furthermore, we report a uniquely high abundance of several *Bifidobacterium* species in multiple residents and excluded the possibility that this was due to probiotic supplementation.

**Supplementary Information:**

The online version contains supplementary material available at 10.1186/s13073-021-00869-z.

## Background

Infections caused by multidrug-resistant organisms (MDROs) are a rising threat to global health and caused ~ 33,000 attributable deaths in Europe in 2015 [1]. Infections with MDROs are usually preceded by asymptomatic gut colonisation, and asymptomatically colonised individuals represent a potential transmission reservoir [2]. Nursing home residents are at increased risk for MDRO colonisation due to comorbidities resulting in increased healthcare contact and antibiotic use [3]. In addition, MDRO spread within a nursing home can be facilitated due to communal living, confined living space and incontinence of residents [4, 5]. This is similar to the transmission dynamics of *Clostridioides difficile*. The prevalence of MDROs and *C. difficile* varies between nursing homes from different countries, but large differences in prevalence can also be observed between different institutions in one country. For example, MDRO prevalence ranges from 0 to 47% in various nursing homes in the Netherlands [6–8] and from 0 to 75% in Ireland [5]. *C. difficile* colonisation prevalence ranges from 0 to 17% in Dutch nursing homes [9, 10] and from 0 to 10% in Germany [11]. These differences may reflect variation in individual nursing home infection prevention and control practices, antimicrobial stewardship, infrastructure, care load and presence of MDRO risk factors such as incontinence, recent hospitalisation and current antibiotic use. Colonisation resistance provided by the gut microbiome could contribute to preventing MDRO colonisation in the gut. The gut microbiome can provide colonisation resistance through secretion of antimicrobial products, nutrient competition, support of epithelial barrier integrity, bacteriophage deployment and immune activation. However, current knowledge on the link between the microbiome and MDRO colonisation is limited [12, 13]. In travellers, an increase of antimicrobial resistance genes and *Escherichia coli* relative abundance in the microbiome were observed after acquisition and asymptomatic carriage of extended-spectrum beta-lactamase (ESBL)-producing *E. coli*, but without clear differences in microbial community structure [14]. An exception to the understudied role of the microbiome in MDRO colonisation is vancomycin-resistant *Enteroccocus* (VRE). For example, it has recently been demonstrated that a lantibiotic-producer, in this case *Blautia producta*, could restore colonisation resistance against VRE [15].

To determine the prevalence and spread of MDROs in a Dutch nursing home, and to elucidate the role of the gut microbiota and clinical risk factors herein, we conducted a four-point-prevalence study and analysed clinical data of residents and whole-genome sequencing (WGS) data of MDRO isolates, in combination with gut microbiota analysis through 16S rRNA gene amplicon sequencing. In addition, we conducted more in-depth microbiota analysis in a selection of samples through metagenomics in order to further investigate findings from 16S rRNA gene amplicon analysis.

## Methods

### Study design

We conducted a prospective cohort study in which residents of a nursing home in the Netherlands were invited to participate. The prevalence, dynamics and risk factors of MDRO colonisation were studied in a non-outbreak situation. Demographical, epidemiological and clinical data of four time points with a 2-month interval (October 2016 until April 2017) were collected. Microbiota analysis was performed on stool samples collected at the same four time points. Written informed consent was obtained from the resident or corresponding proxy. Ethical approval was granted by the medical ethics committee of the Leiden University Medical Center (No.P16.039). Sixty-four of 131 residents (49%) consented to participate. Data and corresponding faeces were collected from 60 residents (94%). To make optimal use of the longitudinal data from this study, residents were selected whom provided faeces at at least two time points (*n* = 47). For this study, we included residents who gave consent for additional analyses, from whom faeces were cultured for MDROs at at least two time points, and of which sufficient material was left for microbiota profiling at at least two time points (*n* = 27 residents). The prevalence of MDRO was not statistically significant between the residents selected for microbiota analysis (12/27 residents and 27/93 time points) and those not selected (10/30 residents and 12/61 timepoints) (chi-squared test, *p* = 0.26).

### Data and faeces collection

The nursing home consisted of 131 beds divided over eight wards of various sizes (12–35 beds). The wards had single en-suite rooms, except for three double rooms for couples. All wards had a separate dining area where freshly prepared meals were served daily and residents did not receive a specific diet or probiotics. In addition, the nursing home had a large communal recreation and shared physiotherapy area. Nursing staff was dedicated to specific wards, but occasionally staff cross-covered wards. For each consenting resident, socio-demographic and the following MDRO risk factor data were collected at each of the four time point using standardised ECDC definitions: care load indicators (disorientation, mobility, incontinence), hospitalisation in the previous 6 months, antibiotics (concomitant and in the previous 6 months), comorbidities, presence of an indwelling urinary catheter or wounds and history of past MDRO colonisation [16].

In addition, instructed caring staff collected fresh faeces on the four time points and subsequently stored the samples at 4 °C. Samples were transported within 72 h to the laboratory (Leiden University Medical Center).

### MDRO detection

Faecal samples were examined for multi-drug-resistant bacteria by culturing within 8 h after arrival at the laboratory and the faeces and cultured MDROs were subsequently stored at − 20 °C [9]. Based on national recommendations [17], the following micro-organisms were considered to be an MDRO: ESBL-producing *Enterobacterales*; *Enterobacterales* and *Acinetobacter spp.* resistant to both fluoroquinolones and aminoglycosides or carbapenemase-producing; carbapenemase-producing *Pseudomonas aeruginosa*; *P. aeruginosa* resistant to at least three of the following antibiotic classes: fluoroquinolones, aminoglycosides, ceftazidime and/or piperacillin; trimethoprim/sulfamethoxazole-resistant *Stenotrophomonas maltophilia*; or vancomycin-resistant enterococci (VRE). Faecal samples were enriched in 15 ml of Tryptic Soy Broth (TSB) and incubated for 18 h at 35 °C prior to plating on ChromID ESBL, ChromID VRE and MacConkey tobramycin agars (BioMérieux, Marcy l’Etiole, France) for 48 h at 35 °C [9]. The twenty samples of the first time point were re-cultured 2 years after sampling, as these samples were initially enriched with TSB containing 8 mg/L vancomycin and 0.25 mg/L cefotaxime. The samples were stored in − 20 °C with glycerol. All morphological different aerobic Gram-negative bacteria and enterococci were identified by the BD Bruker matrix-assisted laser desorption ionisation-time of flight (MALDI-TOF) Biotyper (Microflex, Bruker Daltonics, Bremen, Germany). Phenotypic antibiotic susceptibility testing was performed with the VITEK2 system (card N199, BioMérieux) using the European Committee of Antimicrobial Susceptibility Testing (EUCAST) breakpoints [18]. ESBL production was confirmed by a double-disk method [19]. In addition, the faecal samples were screened for the presence of carbapenemase-producing Gram-negative bacteria [19]. The minimum inhibitory concentration (MIC) of *Enterobacterales* with a meropenem MIC > 0.25 mg/L was confirmed with an antibiotic gradient strip method (Etest, BioMérieux). Strains with an MIC > 0.25 mg/L were further investigated by an in-house multiplex PCR to detect the most frequently found carbapenemase genes (KPC, VIM, NDM, OXA-48 and IMP). Additionally, *Clostridioides difficile* was cultured and characterised as previously described [20].

### Risk factor analysis

Data from 27 nursing home residents (93 samples in total) were included for risk factor analysis. All analyses compared all MDRO-positive samples with all MDRO-negative samples, as extensive metadata was collected at each time point for each individual resident. To account for the repeated measurements design, generalised estimating equations (GEE) logistic regressions (using the geeglm() function in the geepack package) were performed with Resident number as cluster [21]. To identify clinical factors associated with MDRO colonisation, univariable GEE logistic regression was performed using variables for which ten or more ‘events’ were recorded, as previously recommended for logistic regression [22]. Factors with a *p*-value < 0.05 were included in multivariable GEE logistic regression analysis, as well as non-significant factors that were considered likely to influence MDRO colonisation risk based on expert opinion and literature review. These factors were sex and current use of a urinary catheter. Lastly, we inspected possible multicollinearity between the variables included in the multivariable GEE logistic regression by computing variance inflation factors. While opinions differ on when a variance inflation factor can be considered considerable, we used the stringent variance inflation factor value of 2.5 here, as previously recommended, to obtain insight in possible multicollinearity [23].

### Whole-genome sequencing of bacterial isolates and data processing

WGS analysis to characterise MDRO isolates was done at GenomeScan B.V. (Leiden, the Netherlands). Genome sequences were determined using the Illumina HiSeq 4000 platform (Illumina, San Diego, CA, USA) from DNA prepared by the QIAsymphony DSP Virus/Pathogen Midi Kit (Qiagen, Hilden, Germany) at Leiden University Medical Center following manufacturer’s recommendations. Sequence libraries were prepared using NEBNext® Ultra™ II DNA Library Prep Kit for 150-bp paired-end sequencing.

Sequencing quality was evaluated with FastQC (version 0.11.8) [24] and MultiQC (version 1.7) [25]. Reads were assembled using a hybrid assembly strategy, starting with SKESA (version 2.3.0) [26] using default parameters for paired-end reads, followed by SPAdes (version 3.13.1) [27] using default parameters while providing SKESA’s contigs with the ‘--untrusted-contigs’ parameter. Assembly quality and length were checked after each step using QUAST (version 5.0.2) [28]. The scaffolds produced by SPAdes were used for subsequent analyses.

To evaluate assembly quality, all scaffolds were blasted (megablast version 2.9.0, parameters ‘-evalue 1e-10’ and ‘-num_alignments 50’) [29, 30] against the NCBI BLAST nt database (from July 13, 2017) and taxonomically classified using the Lowest Common Ancestor algorithm implemented in Krona ktClassifyBLAST (version 2.7.1) [31]. Scaffolds classified as eukaryote were removed from further analysis. The remaining non-eukaryotic scaffolds were screened for the presence of antibiotic resistance genes using staramr (version 0.5.1, https://github.com/phac-nml/staramr) and ABRicate (version 0.8.13, https://github.com/tseemann/abricate) against the ResFinder database (from May 21, 2019) [32]. The same scaffolds were also subjected to in silico multi-locus sequence typing (MLST) and core-genome MLST using SeqSphere (version 6.0.2, Ridom GmbH, Münster, Germany) [33] to determine Warwick sequence types (ST) and pairwise allele distances using the built-in *E. coli* scheme. Next, a pangenome analysis was conducted on the scaffolds using Roary (version 3.12.0) [34], for which the scaffolds were annotated using Prokka (version 1.13.4) [35]. Finally, a maximum-likelihood phylogenetic analysis was generated with IQTree (version 1.6.10, parameters ‘-b 500’ and ‘-m MFP’ for 500 bootstrap replicates and automatic model selection) [36] on the multiple sequence alignment of the core genomes generated by Roary. The selected phylogenetic model based on the best Bayesian Information Criterion score was GTR+F+R2.

All tools were run with default parameters unless stated otherwise.

### DNA extraction for gut microbiota analyses

DNA was extracted from 0.1 g faeces (*n* = 93 samples) using the Quick-DNA™ Fecal/Soil Microbe Miniprep Kit (ZymoResearch, CA, USA) according to manufacturer’s instructions with minor adaptations, as described previously [37]. Beads were a mix of 0.1 and 0.5 mm size, and bead-beating was performed using a Precellys 24 tissue homogeniser (Bertin Technologies, France) at 5.5 m/s for three times 1 min with short intervals.

### 16S rRNA gene amplicon sequencing

Quality control, library preparation and sequencing were performed by GenomeScan B.V. (Leiden, The Netherlands) using the NEXTflex™ 16S V4 Amplicon-Seq Kit (BiooScientific, TX, USA) and the Illumina NovaSeq6000 platform (paired-end, 150 bp). Raw reads were processed using the NG-Tax 0.4 pipeline with following settings: forward read length of 120, reverse read length of 120, ratio OTU abundance of 2.0, classify ratio of 0.9, minimum threshold of 1 × 10^−7^, identity level of 100% and error correction of 98.5, using the Silva_132_SSU Ref database [38, 39]. Since a 100% identity level was used, amplicon sequence variants (ASVs) were obtained. The obtained ASV table was filtered for ASVs with less than 0.005% relative abundance [40]. Three ZymoBiomics Microbial Community Standards (Zymo Research, Irvine, CA, USA), two ZymoBiomics Microbial Community DNA Standards (Zymo Research) and three negative DNA extraction controls were included as positive and negative controls for DNA extraction and sequencing procedures.

### Metagenomic sequencing

Ten faecal samples (two samples from five residents) and two positive controls were selected for metagenomic shotgun sequencing. Quality control, library preparation and sequencing were performed by GenomeScan B.V. (Leiden, The Netherlands) using the NEBNext® Ultra™ II FS DNA Library Prep Kit (New England Biolabs, Ipswich, Massachusetts, USA) and the Illumina NovaSeq6000 platform (paired-end, 150 bp). Raw shotgun sequencing reads were processed using the NGLess (v1.0.1) language and accompanying tools [41–45]. NGLess is a domain-specific language especially designed for processing raw sequence data and designed for enabling user-friendly computational reproducibility. Pre-processing of raw data was performed as previously described [41]. In short, raw sequence data was first pre-processed by performing quality-based trimming and reads with quality value below 25 were discarded, followed by discarding reads shorter than 45 bp. Second, reads were aligned to the human genome (hg19 reference) and discarded if reads mapped with more than 90% sequence identity and an alignment length of at least 45 bp. Third, taxonomic profiling was performed using the mOTUs2 (v2.5.1) tool using default parameters as previously described [44]. This profiler is based on ten household, universal, single-copy marker gene families and profiles bacterial species both with (ref-mOTUs) and without (meta-mOTUs) a sequenced reference genome. A relative abundance table was obtained as output.

Next to the read-based analysis described above, we used an assembly-based analysis pipeline, Jovian (version v0.9.6.1) [46]. In short, the pipeline checks read quality, trims low-quality reads, removes reads derived from the host organism (human) and de novo assembles reads into scaffolds which are then taxonomically classified and quantified. These classifications were used to support the read-based results and scaffolds of selected species were compared to one another using pyANI (version 0.2.10) to calculate pairwise average nucleotide identities [47].

### Positive and negative controls for gut microbiota profiling

#### Included controls indicate good DNA extraction and sequencing performance

An average of 24,095 reads (range 4841–68,057, median 22,775 reads) was generated per sample for 16S rRNA gene amplicon sequencing (total *n* = 93), resulting in 1042 ASVs after filtering on 0.005% abundance. Both positive DNA sequencing controls (*n* = 2) were highly similar to theoretical expectations (average fold change 1.11), while DNA extraction controls (*n* = 3) were somewhat less similar to theoretical expectation (average fold change 1.81). One DNA extraction control showed a lower than expected abundance (~ 12 fold) of *Staphylococcus* for unknown reasons (Additional file 1: Fig. S1A). Of the three included negative extraction controls, two did not contain any reads post-filtering and one negative control contained 21 reads, mostly from known contaminants such as *Delftia* and *Streptococcus*, as previously observed [37].

For metagenomic sequencing, the DNA extraction control and sequencing control closely matched theoretical profiles and eight mOTUs were identified, apart from a small fraction of unassigned reads (Additional file 1: Fig. S1B).

### Statistical analysis and visualisations

Analyses and visualisations were performed in R (v3.6.1), using the following packages: phyloseq (v1.28.0), microbiome (v1.6.0), Metalonda (v1.1.5), DESeq2 (v1.24.0), tidyverse packages (v1.2.1), pheatmap (v1.0.12) and ggplot2 (v3.2.0) [48–54].

#### Community composition analysis

Permutational multivariate analysis of variance (PERMANOVA) using Bray-Curtis dissimilarity was performed to test for differences in overall community composition. Prior to employing PERMANOVA testing, it was tested whether groups had homogenous dispersions (homoscedasticity) using the betadisper function, as violation of this statistical assumption can lead to erroneous conclusions regarding PERMANOVA results. No heteroscedasticity was observed between groups. To account for the repeated measurements design, we used ‘strata=Resident number’. Principal coordinates analysis (PCoA) based on Bray-Curtis dissimilarity was made and 95% confidence intervals were computed using the stat_ellipse function. Alpha diversity indices (observed ASVs/observed genera and Shannon index) were compared using independent *t*-tests or Wilcoxon rank sum tests. For calculating intraindividual stability, Bray-Curtis dissimilarities between all samples of a resident were calculated, and this was averaged to obtain a mean stability per resident.

#### Differential abundance analysis

Differential abundance analysis between groups (MDRO-positive samples versus MDRO-negative samples) was performed at genus level using DESeq2 and stratified per time point. Genera had to be present in at least 25% of samples to be included in the analysis. To correct for false discovery rate, *p*-values were corrected using the Benjamini-Hochberg procedure. Considering the low number of MDRO-positive samples per time point, adjusted *p*-values < 0.1 were included in visualisation of results.

#### Time series modelling of alpha diversity

Linear mixed models were applied to investigate the changes in alpha diversity over time between the ever colonised versus never colonised groups using the lme4 and lmerTest packages [55, 56]. Ever colonised was defined as having an MDRO-positive sample at at least one time point during the study, while never colonised was defined as having no MDRO-positive sample during the study. Resident number was included as a random intercept to control for inter-individual baseline differences and repeated measurements design. The included fixed effect was the interaction between ‘ever colonised’ and timepoint (‘ever colonised’*timepoint). Models were inspected for normally distributed residuals using qq-plots and *p*-values < 0.05 were considered significant.

#### Time series modelling of individual taxa

To identify temporal trends in differential abundance of bacterial genera, the metagenomic longitudinal differential abundance method (MetaLonDA) package was used [50]. Only residents with at least three available gut microbiota samples were included in this analysis (*n* = 24 residents). Genera had to be present in at least 25% of samples to be included in the analysis. MetaLonDA is capable of handling inconsistencies often observed in human microbiome studies (e.g. missing samples) and relies on two main modelling components, the negative binomial distribution for modelling read counts and smoothing spline ANOVA for modelling longitudinal profiles. The function metalondaAll was used with the following settings: n.perm=1000, fit.method=“nbinomial”, num.intervals=3, pvalue.treshold=0.05, adjust.method=“BH”, norm.method=“median_ratio”. These settings indicate that the function was run with 1000 permutations using the median ratio method to normalise count data and fitting a negative binomial distribution. *P*-values were corrected using the Benjamini-Hochberg procedure.

## Results

### Clinical risk factor analysis for MDRO colonisation

#### MDRO colonisation among nursing home residents is highly prevalent and dynamic over time

**Fig. 1:**
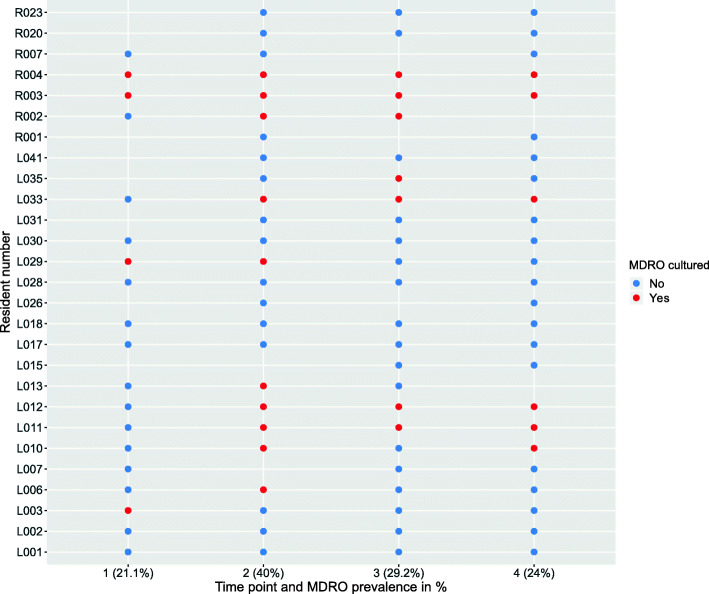
Overview of MDRO status for all samples of each resident over time. Blue colour indicates a negative MDRO culture, while red indicates a positive MDRO culture. Prevalence per time point is shown in percentage. Resident numbers are preceded by either ‘R’ or ‘L’; these letters indicate two physically separated buildings

Of the 27 included residents, twelve (44.4%) were colonised by an MDRO at at least one time point; four (33.3%) were colonised at one time point and eight residents (66.7%) at more than one time point during the 6-month study (Fig. 1). Of the 93 faecal samples, 27 (29.0%) contained an MDRO. Fourteen samples (15.1% of all samples) from six different residents (22.2% of all residents) were positive for ESBL-producing bacteria, of which ten were *E. coli*, three *Enterobacter cloacae* and one *Citrobacter* non*-koseri*. The remaining thirteen MDRO isolates (14.0% of all samples) were both fluoroquinolone- and aminoglycoside*-*resistant *E. coli.* No carbapenemase-producing Gram-negative bacteria, VRE and *Clostridioides difficile* were cultured. As MDROs in the current study are exclusively MDR *Enterobacterales*, we refer to MDR *Enterobacterales* as MDROs from here onwards.

#### Clinical risk factors are only associated with MDRO colonisation in univariable analysis

Analysis of MDRO-status of faecal samples and clinical data using univariable GEE logistic regression showed several factors related to an increased risk of MDRO colonisation, including bone fracture in medical history (*p* = 0.031, odds ratio (OR) 4.39, 95% confidence interval (CI) 1.14–16.95), antibiotic use in the past 2 months (*p* = 0.039, OR 3.06, 95% CI 1.06–8.85) and hospital admittance in the last year (*p* = 0.043, OR 4.95, 95% CI 1.05–23.34). Based on expert opinion, we further included sex and present use of urinary catheter as variables in multivariable GEE logistic regression. After including all variables in a multivariable GEE logistic regression only antibiotic use in the past 2 months displayed a trend (*p* = 0.088, OR 2.84, 95% CI 0.85–9.49), while hospital admittance in the past year (*p* = 0.13, OR 3.78, 95% CI 0.69–20.70) and bone fracture in medical history (*p* = 0.35, OR 1.95, 95% CI 0.48–8.00) became non-significant. Lastly, multicollinearity between the included variables was assessed by computing variance inflation factors, but no considerable collinearity was observed (variance inflation factors for all variables < 2).

### WGS of bacterial isolates

As most isolated MDRO strains were *E. coli* strains (22/27, 81.5%), we focused our analyses on this species. The 22 isolates were derived from 11 residents and were analysed by whole-genome analysis, including maximum likelihood phylogeny of core genes, accessory genome clustering, core-genome MLST and profiling of antibiotic resistance genes.

#### Genome-based clustering reveals a ward-specific *E. coli* ST131 strain

**Fig. 2:**
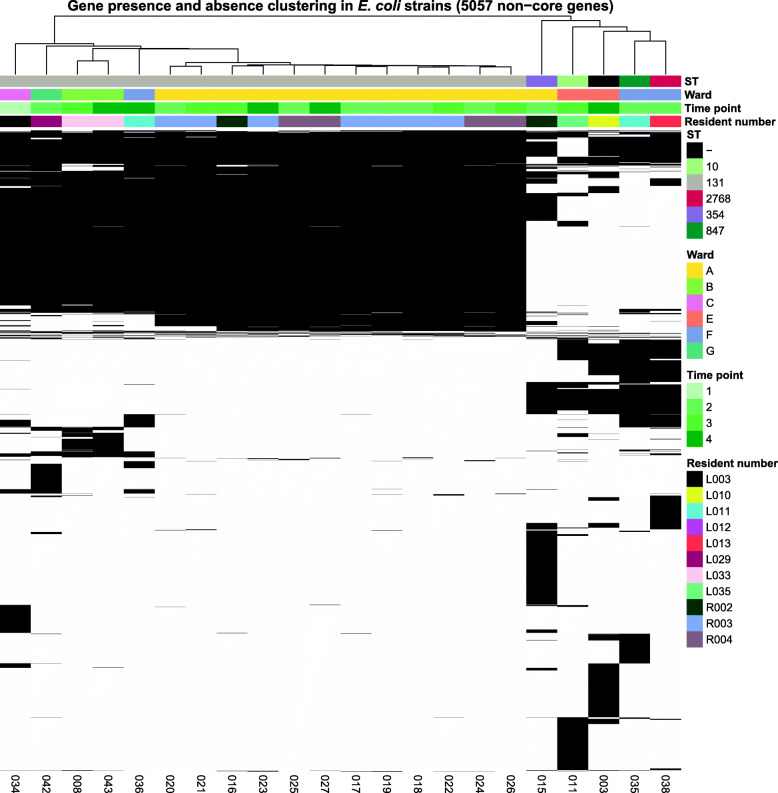
Overview of the accessory genome (non-core genes) of the 22 *E. coli* strains from eleven residents at different time points. Accessory genes are clustered based on the average linkage method using Euclidean distances. All (*n* = 17) ST131 isolates cluster together, while the other STs form a separate cluster. In addition, ST131 from ward A cluster together and are different from ST131 from other wards. The y-axis displays accessory genes and the *x*-axis isolate numbers. Black bars indicate presence and white bars absence of a gene

Based on pangenome analysis, we identified core and accessory (non-core) genes, of which the accessory genes (5057) were selected for clustering. Clustering based on presence/absence of these accessory genes showed a clear cluster of ST131 strains (Fig. 2). Within the ST131 cluster, two separate clusters could be observed, one closely related cluster of twelve isolates belonging to three residents on ward A, and one cluster of four less related isolates from four residents of four different wards. The isolates of three residents on ward A (R002, R003 and R004) have nearly identical accessory genes, suggesting that they were colonised with the same strain. In addition, these isolates have a nearly identical accessory genome over time, suggesting persistent colonisation of the same strain. Clustering based on the maximum likelihood phylogeny of core genes also resulted in a clear clustering of ST131 strains (data not shown). In addition, while the differences are smaller than in the accessory genome, ST131 strains from ward A still cluster separately from ST131 strains from other wards. Lastly, a core-genome MLST confirms clustering of ST131 strains on ward A (with up to two alleles difference) and shows that ST131 isolates from other wards are different (with more than 30 alleles difference) (Additional file 1: Fig. S2). These results support the hypothesis that an ST131 strain was spread across ward A.

#### Specific resistance genes are exclusive to certain wards

**Fig. 3:**
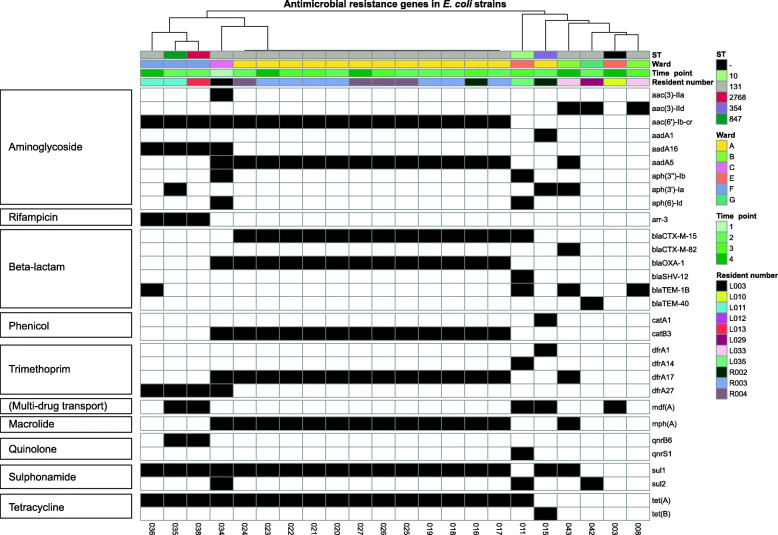
Heatmap of antibiotic resistance genes in the 22 *E. coli* isolates from eleven residents at different time points. Black boxes indicate presence of a resistance gene, while white indicates absence of the resistance gene. Antibiotic resistance gene profiles are clustered by hierarchical clustering using Euclidian distances. Resident number, time, ward and time point are given as coloured annotations

Next, the prevalence of antibiotic resistance genes was determined. Based on resistance gene absence/presence in the genome, ST131 largely clustered together (Fig. 3), and again a cluster of ST131 belonging to residents of one ward (ward A) was observed. These strains were characterised by presence of nine resistance genes (*aac (6’)-Ib-cr*, *aadA5*, *bla-CTX-M-15*, *blaOXA-1*, *catB3*, *dfrA17*, *mph(A)*, *sul1* and *tet(a)*). Three isolates belonging to ST131, 847 and 2786 from ward F clustered together, and these three strains (from two residents) contained the rifampicin resistance gene *arr-3*, which was not detected in other strains.

### Gut microbiota analysis using 16S rRNA gene amplicon sequencing

#### A distinct gut microbiota between MDRO-positive and MDRO-negative samples

**Fig. 4:**
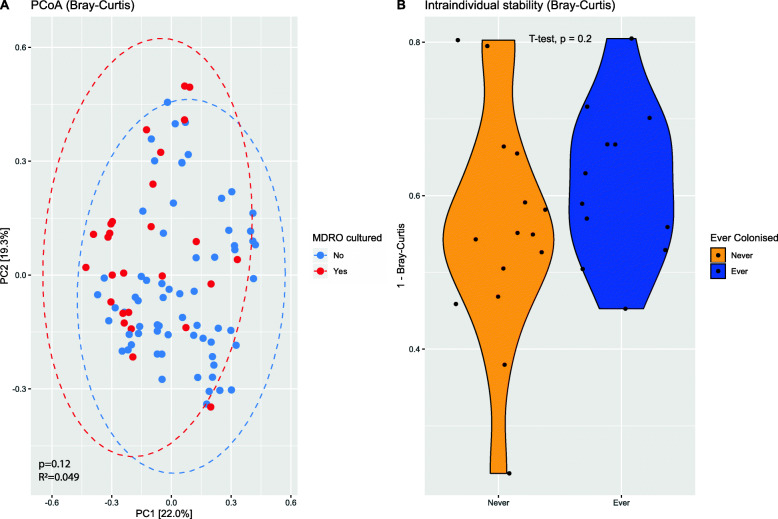
Bray-Curtis distance measures visualised by principle coordinates analysis (PCoA) for all (*n* = 93) faecal samples based on whether an MDRO was cultured (**a**) and by mean intraindividual stability (1 - Bray-Curtis dissimilarity) between ‘ever’ and ‘never’ colonised residents (**b**). Each dot in plot A represents a single sample, and ellipses indicate 95% confidence intervals

First, alpha diversity (using observed ASVs/genera and Shannon index) was computed at both ASV and genus level to compare MDRO-positive with MDRO-negative samples. To account for repeated measures, we stratified these alpha diversity analyses by time point. No significant differences in alpha diversities at either level at any time point were observed (Additional file 1: Fig. S3). Beta diversity was also not significantly different between these samples (*p* = 0.12 and *R*^2^ = 0.049) (Fig. 4a). To identify individual bacterial taxa associated with MDRO status, differential abundance analysis was performed using DESeq2 at each time point. Several taxa were more abundant in MDRO-negative samples on multiple timepoints, namely *Atopobiaceae*, *Coprococcus_3*, *Dorea*, *Enorma*, *Holdemanella*, *Lachnospiraceae*, *Lachnospiraceae_ND3007_group*, *Phascolarctobacterium* and *Ruminococceae_UCG-014* (Additional file 1: Fig. S4, Additional file 2: Table S1). Only three taxa (*Erysipelatoclostridium, uncultured_Coriobacteriales* and *uncultured_Ruminococcaceae)* were more abundant in MDRO-positive samples at any time point.

#### MDRO colonisation is associated with consistent differences in relative abundance of specific bacterial taxa

Residents and their samples were further classified on having been MDRO-colonised at at least one time point during the study (ever, *n* = 45 samples) or not (never, *n* = 48 samples). There were no differences in alpha diversities over time between the groups (Additional file 1: Fig. S5), nor in beta diversity (intra-individual stability) between the ever and never colonised group (independent t-test, *p* = 0.2) (Fig. 4b).

**Fig. 5:**
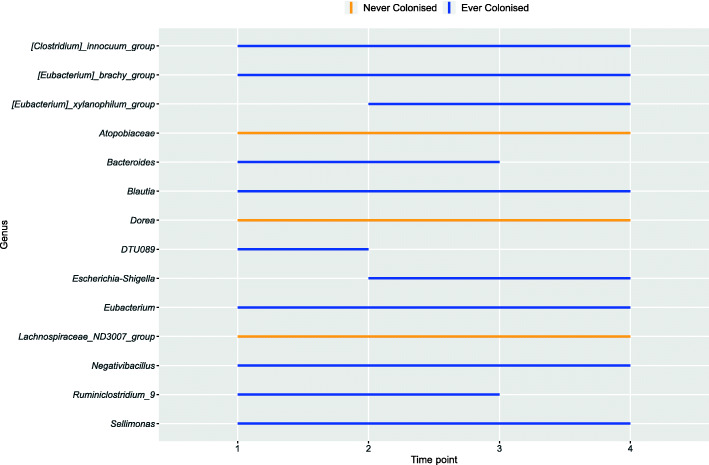
Time intervals of significantly different bacterial genera between ever (*n* = 12) and never (*n* = 15) MDRO colonised residents. Each line interval represents a significant time interval, with significance being considered *p* < 0.05. Orange lines indicate higher abundance in the never colonised group, while blue indicates higher abundance in the ever colonised group. If no coloured line is observed, the respective genus is not significantly differentially abundant between specific time points

Longitudinal differential abundance analysis between samples from ‘ever’ versus ‘never’ MDRO-colonised residents was performed to investigate whether differences in relative abundance were consistent over time. From each resident, at least three out of four samples should have been available to be included in this analysis, resulting in 45 samples from ever colonised residents and 42 samples from never colonised residents. Three taxa (*Atopobiaceae*, *Dorea* and *Lachnospiraceae_ND3007_group*) were consistently more abundant in ‘never’ colonised residents throughout the 6-month study period (Fig. 5, Additional file 1: Fig. S6). These taxa were also identified to be more abundant in MDRO-negative samples compared to MDRO-positive samples at two time points (Additional file 1: Fig. S4).

Lastly, we looked for intra-individual changes in pairs of samples of residents who either became MDRO colonised or were MDRO decolonised during the study period. For this, samples were analysed of an MDRO-negative sample prior to an MDRO-positive sample (*n* = 8 residents), and vice versa; an MDRO-positive sample followed by an MDRO-negative sample (*n* = 6 residents). Resident L10 could be included twice in the first comparison, but to avoid excessive impact of this resident on statistical analysis, it was included once. We then performed paired analyses for each of the two groups. However, no differences in alpha or beta diversity were observed, nor were any genera differentially abundant in any of the comparisons (data not shown).

#### Compositional profiles show very high abundance of *Actinobacteria* members *Bifidobacterium* and *Collinsella*

**Fig. 6:**
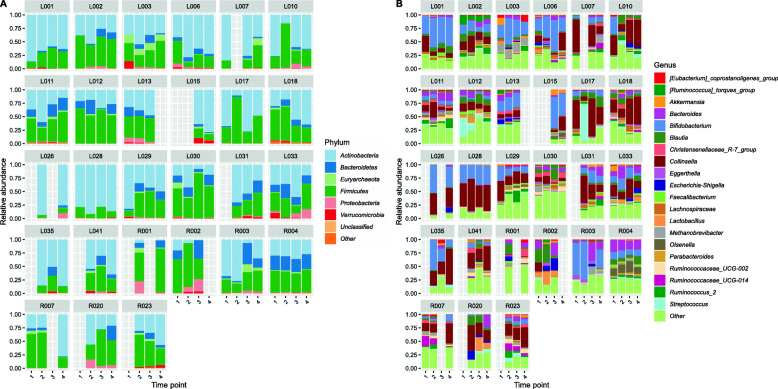
Compositional profiles at phylum level (**a**) and genus level (**b**) from 16S rRNA gene amplicon data of 27 residents at four time points. Other indicates the sum of all bacterial phyla or genera not specifically indicated in the legend. The *y*-axis displays relative abundance and the *x*-axis the study time point

Next, we investigated the global microbiota profiles across all residents without a focus on MDRO colonisation. Compositional profiles at phylum and family level showed that the most abundant phylum in multiple residents was *Actinobacteria* (Fig. 6a), which is in contrast to what is considered a ‘normal’ gut microbiota that generally consists of ~ 90% *Firmicutes* and *Bacteroidetes. Bifidobacterium* and *Collinsella* were the *Actinobacteria* members with highest relative abundance (Fig. 6b).

### Metagenome analysis using shotgun sequencing data of ten faecal samples

#### Not a single species, but several *Bifidobacterium* species are highly abundant in residents

**Fig. 7:**
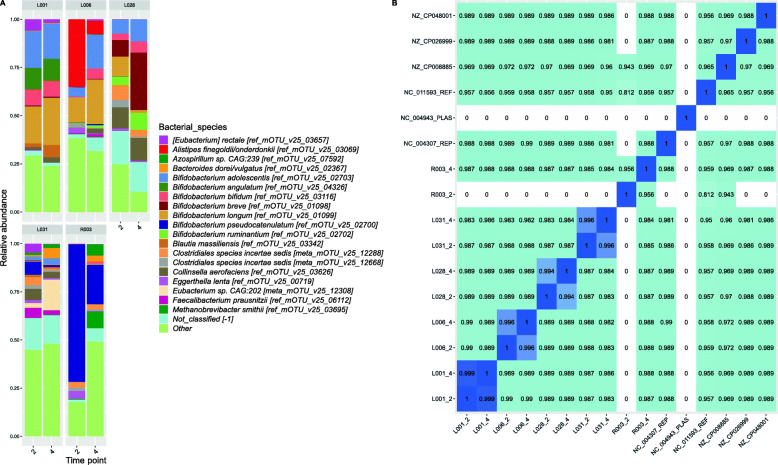
Compositional plot based on metagenomes of ten faecal samples from five residents using mOTUs (**a**) and average nucleotide identity between assembled *B. longum* strains and reference sequences (**b**). Relative abundance is shown of the twenty most abundant bacterial species in all samples and different bacterial species are indicated by colours. ‘Other’ is the sum of the relative abundance of all species not listed in the colour key. Numbers on the *x*-axis indicate the resident number and study time point. Average nucleotide identity of *B. longum* strains as computed by pyANI. The sequence labelled ‘NC_004307_REP’ in B is the representative genome on GenBank; the sequence with ‘NC_004943_PLAS’ is its plasmid. The sequence with ‘NC_011593_REF’ is the *B. longum* reference genome

The nursing home did not provide probiotics to their residents. However, the high abundance of *Bifidobacterium* in the residents’ stools suggested otherwise. Ten stool samples from five residents with high *Bifidobacterium* and/or *Collinsella* relative abundance were further investigated by shotgun metagenomic sequencing, and two positive controls were included. The high relative abundance of *Bifidobacterium* and *Collinsella* could be confirmed and residents were colonised by seven highly abundant *Bifidobacterium* species, namely *B. adolescentis*, *B. angulatum*, *B. bifidium*, *B. breve*, *B. longum*, *B. pseudocatenulatum* and *B. ruminantium* (Fig. 7a). From these species, *B. adolescentis*, *B. bifidum*, *B. breve* and *B. longum* are the most commonly used species in probiotics, although the others have been studied for probiotic properties as well [57].

#### Assembly-based method reveals that *Bifidobacterium longum* strains are (almost) identical within residents, but not between residents

To investigate whether *Bifidobacterium longum* strains were identical between and within residents, we analysed the strains using de novo assemblies. *B. longum* was selected because of its high relative abundance in multiple samples, increasing the chance of recovering a full genome from the respective metagenomes and because it is commonly present in probiotics. Its genome size is about 2.5 Mb and contains a high GC content of ~ 60%. From samples of residents L001, L006 and L028, *B. longum* genomes larger than 2 Mb could be recovered, indicating that (nearly) full genomes were successfully obtained from the metagenome, but this was not the case for L031 and R003 (Additional file 3: Table S2). While average nucleotide identities were high between samples, strains from the same individual were more identical to themselves than to strains from other residents (Fig. 7b). This indicates that residents do not carry the same *B. longum* strains. It should be noted that a full *B. longum* genome could not be retrieved for all residents. Lastly, *B. longum* genomes were compared to the NCBI reference genome (accession number NC_011593), the representative genome (NC_004307) and its plasmid (NC_004943) and several other *B. longum* strains (Fig. 7b) to provide insight in what levels of divergence are to be expected between strains. Comparing these *B. longum* genomes from the NCBI database shows that unrelated *B. longum* strains have an average nucleotide identity (ANI) of between 0.956 and 0.988. This further confirms that *B. longum* strains between the nursing home residents were different (maximum ANI between strains from different residents 0.99) and that within residents strains were almost identical (ANI > 0.994), in case a nearly full genome could be retrieved.

## Discussion

We present a unique study on asymptomatic gut MDRO (in this study MDR *Enterobacterales*) colonisation in nursing home residents and performed a wide variety of analyses, namely clinical risk factor analysis, WGS of MDRO isolates and 16S rRNA gene amplicon sequencing and metagenomic sequencing of the gut microbiota. We identify possible risk factors for MDRO colonisation, potential spread of MDROs within a ward and microbial signatures associated with MDRO colonisation using 16S rRNA gene amplicon sequencing. Many of the MDRO-associated microbial signatures are consistent over the 6-month time course of this study as shown by longitudinal modelling. Additionally, the unexpectedly high abundance of *Bifidobacterium* abundance in multiple residents was further investigated using metagenomic sequencing. We show that this high abundance is very unlikely to be stemming from probiotic supplementation, as *Bifidobacterium* species and *B. longum* strains differed between residents.

We observed a spread of *E. coli* ST131 within a ward, but not between wards, as the ST131 seemed ward-specific. *E. coli* ST131 was the most commonly found ST in our study, which is in line with previous results showing that this ST is major driver of the current worldwide spread of ESBL-producing *E. coli* [58, 59]. This sequence type is associated with community-acquired infections and older age, and is frequently observed in nursing homes in countries throughout Europe and the USA [7, 60–62]. While ST131 outbreaks are generally seen among and between various nursing homes, we concluded that spread of specific ST131 strains was restricted within wards. However, previous studies may have been limited by methods to characterise ST131, as they characterise strains only with regular MLST (of a limited number of housekeeping genes). By using pangenome analysis, we investigated the genetic differences in detail, allowing for discrimination of the ST131 strains between the wards. We conclude that MDRO transmission within nursing home wards seems to reflect that of household contacts [63]. This small scale MDRO spread was observed in the samples of 27 residents, one could hypothesise higher absolute numbers of related strains if all nursing home residents would have been screened. Not only strains can spread, plasmids are also able to move between different bacterial strains. For instance, three different *E. coli* ST types found at ward F contained *arr-3*, *aadA16* and *dfrA27*. Considering that these three genes are usually encoded on a plasmid [64, 65], it is possible that they spread between ST131 strains on ward F. However, definite conclusions cannot be made based on these results, as only three MDRO strains were detected in ward F.

Novel microbial signatures of MDRO colonisation were identified which could contribute to colonisation resistance against MDROs. Three taxa were consistently more abundant throughout the study in residents never colonised with an MDRO, namely *Dorea*, *Lachnospiraceae_ND3007_group* and *Atopobiaceae*, and these taxa were also found to be more abundant in MDRO-negative samples at two time points. Increased relative abundance of *Dorea* and the *Lachnospiraceae* family has been shown to be associated with colonisation resistance against *Campylobacter* infection [66]. The relative abundance of *Dorea formicigenerans* was identified as a potential pre-liver transplant marker for subsequent MDRO colonisation [67] but another report did not mention *Dorea* as either a protective taxon or a risk factor [13]. While these results are conflicting, there is a possibility that different studies observed effects of different *Dorea* species or strains, which could theoretically have different or opposing effects on MDRO colonisation. Lastly, as clinical variables were not evenly distributed between compared groups, there is a possibility that observed differences in relative abundance of bacterial taxa can partially be attributed to these confounding factors.

We did not observe differences in alpha diversities between the different groups based on MDRO status. This contrasts several reports where MDRO colonisation was associated with a reduced alpha diversity, although conflicting evidence exists [13, 67, 68]. In addition, no difference in beta diversity was observed between the ever and never MDRO-colonised groups, nor between MDRO-positive and MDRO-negative samples. This contradicts findings in liver transplant patients and MDRO colonisation [67]. Conflicting results regarding diversities and microbial signatures could have multiple reasons. First, technical variation induced from the entire workflow starting with sample collection and ending with use of different statistical tools. Second, different MDRO types were studied between the various reports. In the current study, we mainly observed multi-drug-resistant *E. coli*, while two other major studies investigating MDROs and gut microbiota found a larger variety of MDRO types [13, 67]. Considering that microbiome-mediated colonisation resistance is likely to be specific for individual bacterial species and most likely even bacterial strains, further studies should ideally focus on investigating single MRDOs in relation to the gut microbiota. Third, geographical locations of the studied cohorts were different, likely reflecting differences in gut microbiota composition due to varying dietary patterns and other cultural habits.

An unexpectedly high relative abundance of *Bifidobacterium* was observed in several residents in different wards. Such consistently high relative abundances have, to the best of our knowledge, not previously been described in adults or elderly. Incidental reports of an outgrowth of *Bifidobacterium* species in elderly in a long-term care facility have been described [69]. Rowan et al. observed a high relative abundance of *Bifidobacterium* species in two out of eleven elderly subjects (> 15% relative abundance at at least one time point; mainly *B. longum*, *B. breve* and *B. adolescentis*), although potential explanations were not discussed.

It is known that in infancy the gut microbiota is largely dominated by *Bifidobacterium*, but that this high abundance declines with ageing [70]. In addition, elderly mostly harbour *B. longum*, *B.nucleatum, B. pseudonucleatum* and *B. adolescentis*. While we found that these species were indeed among the most abundant, high relative abundances of *B. angulatum*, *B. bifidus*, *B. breve* and *B. ruminantium* were also observed. At first, we hypothesised that high *Bifidobacterium* relative abundance could be stemming from probiotic supplementation used on a voluntary basis by the nursing home residents, despite knowing that probiotics generally do not colonise very successfully [71, 72]. By performing metagenomic sequencing on a subset of samples, we showed this was unlikely to be the case, as different *Bifidobacterium* species were observed between residents. In addition, using strain-resolved metagenomics, we show that *B. longum* strains were different between residents, but likely the same within residents. Our second hypothesis was related to dietary patterns of residents that perhaps a very monotonous diet could stimulate outgrowth of *Bifidobacterium*. However, residents consumed fresh, daily prepared meals according to a normal Dutch diet. It is unclear what the reasons and consequences of this high relative abundance of *Bifidobacterium* are in our residents. In combination with the observation that a high relative abundance of *Bifidobacterium* is not associated with protection against MDRO colonisation, this suggests that probiotics based on the *Bifidobacterium* species in our study may not effectively protect against MDRO colonisation.

This study has several limitations and strengths. First, our sample size and number of MDRO-positive samples was limited, preventing the application of a more extensive epidemiological risk factor analysis. Sample size was also a limiting factor in differential abundance testing between MDRO-positive and MDRO-negative samples per time point. Second, this study focused on a single nursing home and we can therefore not be certain that microbiota profiles are representative for residents of other (Dutch) nursing homes. Especially in light of our unique findings of high relative abundance of *Bifidobacterium* species, profiling the gut microbiota across other nursing homes would be important. Third, some wards had a very limited number of MDRO isolates, which hampered making definite conclusions about MDRO spread in those wards. Lastly, not all residents provided faecal samples on all four time points.

However, this study uses a unique combination of analyses for in-depth understanding of MDRO spread in a nursing home and the relation of MDRO colonisation with residents’ microbiota. The longitudinal nature of our study setup allowed for (1) detection of robust associations between MDRO colonisation and specific microbial taxa, (2) identifying whether colonising MDRO strains were identical over time and (3) comparing *B. longum* strains within and between residents using strain-resolved metagenomics. In addition, the use of various statistical methods for identifying microbial taxa associated with MDRO colonisation further strengthens our findings. Lastly, our finding of high relative abundance of *Bifidobacterium* in multiple residents warrants further investigation and confirmation by other studies.

## Conclusions

Our study provides new evidence regarding the gut microbiota’s potential in providing resistance against MDRO colonisation in a nursing home. Several specific taxa were identified which were consistently more abundant in residents never colonised with an MDRO throughout the 6-month study. Considering that most of the detected MDROs were *E. coli* strains belonging to ST131, it may be especially interesting to test the potentially protective effect of these taxa against *E. coli* ST131. In addition, we report a uniquely high abundance of several *Bifidobacterium* species in multiple residents and excluded the possibility that this was due to probiotic supplementation. While the reasons for, and consequences of this high relative abundance remain unclear, it does suggest that probiotics based on *Bifidobacterium* species observed in our study are highly unlikely to prevent or eradicate MDRO colonisation in the gut of nursing home residents.

## Supplementary Information

**Additional file 1: Figures S1-S6**.

**Additional file 2: Table S1.** Results of DESeq2 analysis on MDRO-positive samples versus MDRO-negative samples per time point. Negative log2FoldChanges in the table indicate that a genus is more abundant in non-MDRO-colonised samples.

**Additional file 3: Table S2.** Retrieved genome length for *B. longum* in bp from resident samples, and genome length of several reference sequences of *B. longum*. For L001, L006 and L028_2 (nearly) full *B. longum* genomes were obtained (length > 2 million bp).

## Data Availability

The sequencing data in this study are available at the European Nucleotide Archive (ENA) under accession number PRJEB37898 (https://www.ebi.ac.uk/ena/browser/view/PRJEB37898) [73]. All data and R code necessary to reproduce analyses and figures from this manuscript can be found at https://github.com/qducarmon/nursing_home_MDRO [74].

## Abbreviations

MDROs: Multidrug-resistant organisms
ESBL: Extended-spectrum beta-lactamase
VRE: Vancomycin-resistant *Enteroccocus*
WGS: Whole-genome sequencing
TSB: Tryptic soy broth
MALDI-TOF: Matrix-assisted laser desorption ionisation-time of flight
EUCAST: European Committee of Antimicrobial Susceptibility Testing
MIC: Minimum inhibitory concentration
GEE: Generalised estimating equations
ST: Sequence type
ASV: Amplicon sequence variant
PERMANOVA: Permutational multivariate analysis of variance
MetaLonDA: Metagenomic longitudinal differential abundance method
MLST: Multi-locus sequence typing
OR: Odds ratio
CI: Confidence interval
ANI: Average nucleotide identity
