# Supplementary material for: Microbiota-associated risk factors for asymptomatic gut colonisation with multi-drug-resistant organisms in a Dutch nursing home
Source: Genome Med. 2021 Apr 7;13:54. doi: 10.1186/s13073-021-00869-z (PMC8028076; doi:10.1186/s13073-021-00869-z)
Supplement: Supplementary file 1 — Additional file 1: Figures S1-S6. [file 13073_2021_869_MOESM1_ESM.pdf]

Additional File 1 - Microbiota-associated risk factors for asymptomatic gut colonisation with multi-drug resistant organisms in a Dutch nursing home

Quinten R. Ducarmon[a,b,*], Elisabeth M. Terveer[a,b,*], Sam Nooij[a,b], Michelle N. Bloem[a,b], Karuna E.W. Vendrik[a,c], Monique A.A. Caljouw[d], Ingrid M.J.G. Sanders[a], Sofie M. van Dorp[a,e], Man C. Wong[a], Romy D. Zwittink[a,b,*], Ed J. Kuijper[a,b,c,*]

*these authors contributed equally

[a]Department of Medical Microbiology, Leiden University Medical Center, Leiden, The Netherlands

[b]Center for Microbiome Analyses and Therapeutics, Leiden University Medical Center, Leiden, The Netherlands

[c]Center for Infectious Disease Control, National Institute for Public Health and the Environment, Bilthoven, The Netherlands.

[d]Department of Public Health and Primary Care, Leiden University Medical Center, Leiden, The Netherlands

[e]Department of Internal Medicine and Geriatrics, Onze Lieve Vrouwe Gasthuis (OLVG Hospital), Amsterdam, The Netherlands

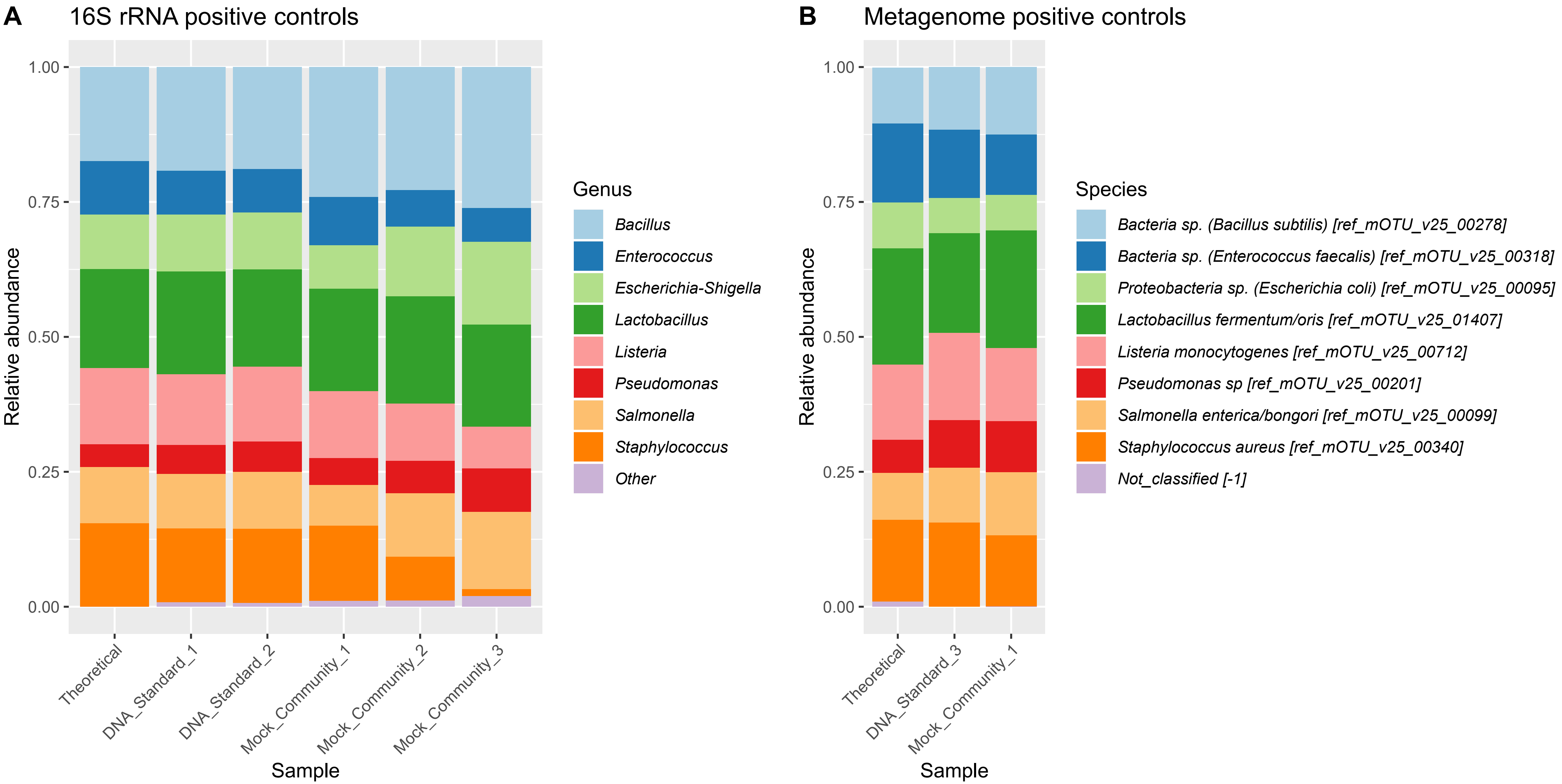

Fig S1: Compositional profiles of positive controls of 16S rRNA gene amplicon sequencing (A) and shotgun sequencing (B). Other indicates the sum of all bacterial genera not specifically indicated in the legend.

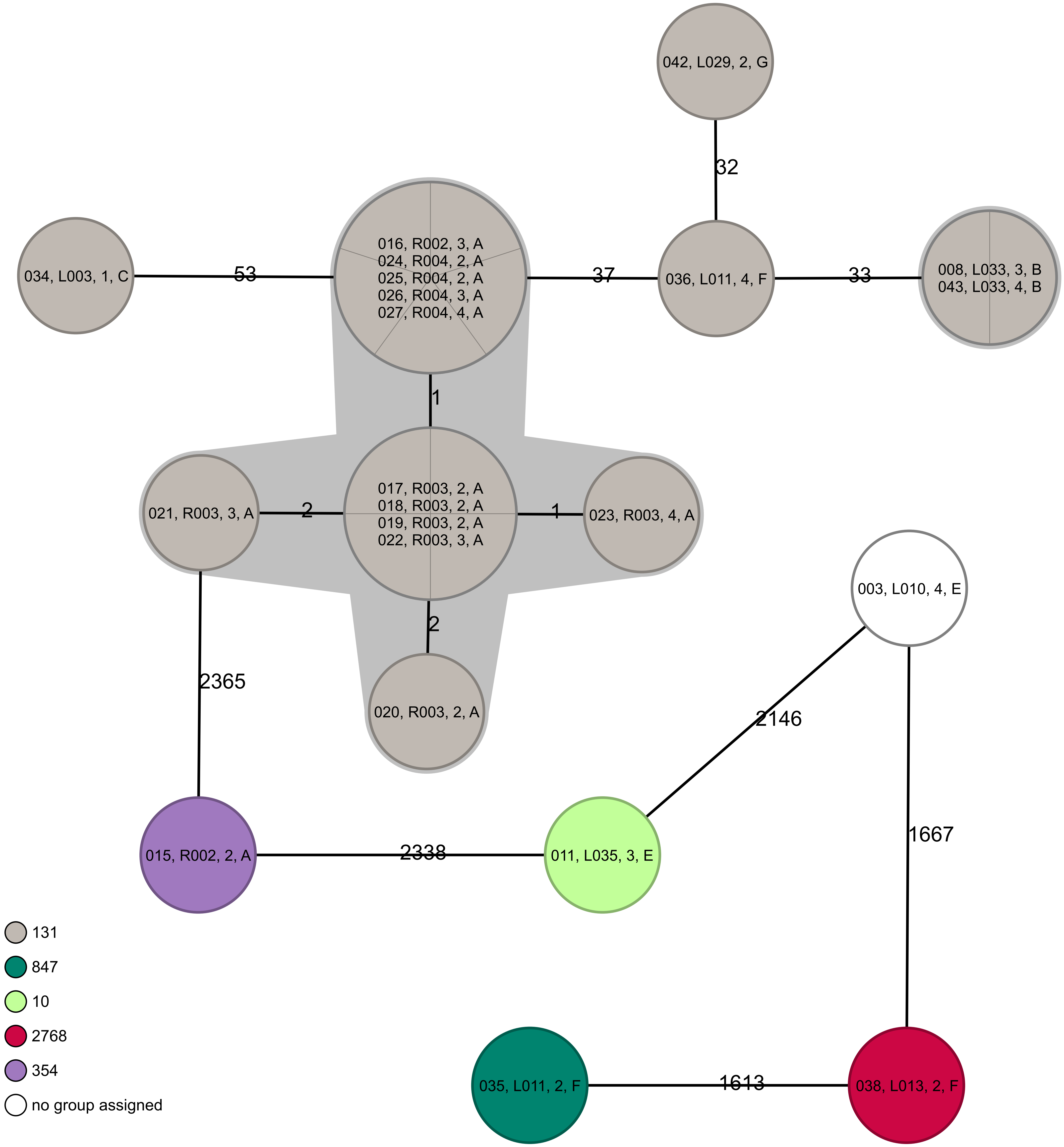

Fig S2: Minimum spanning tree of core-genome MLST (cgMLST) on the 22 E. coli strains. Allelic distances are displayed on edges. Nodes are labelled with isolate number, resident ID, time point and ward. Clusters with distances < 10 alleles are highlighted in grey.

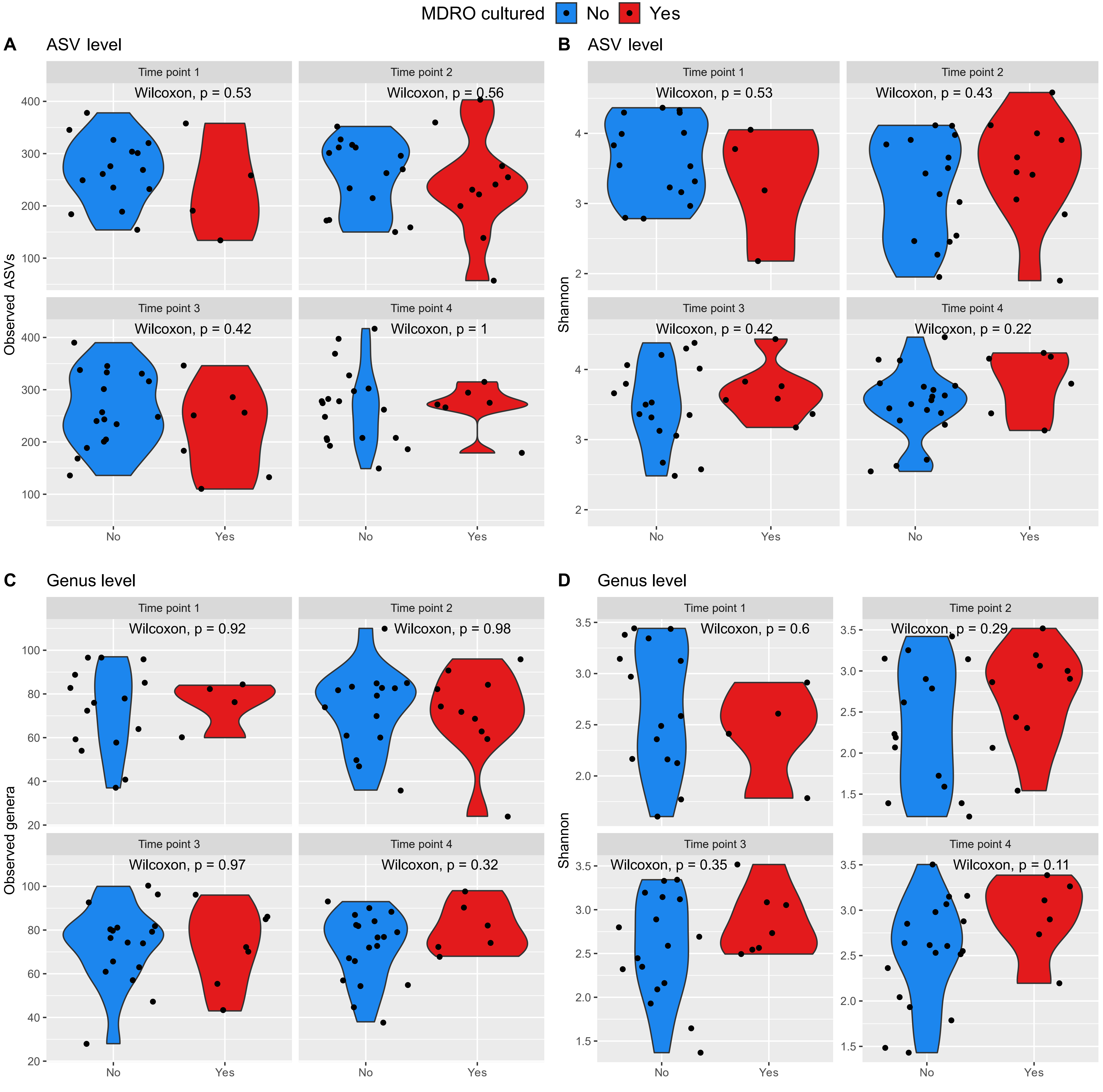

Fig S3: Alpha diversities at ASV (A + B) and genus level (C +D) for samples which had a positive culture for an MDRO versus samples with a negative culture stratified by time point. A Wilcoxon test was performed for each comparison.

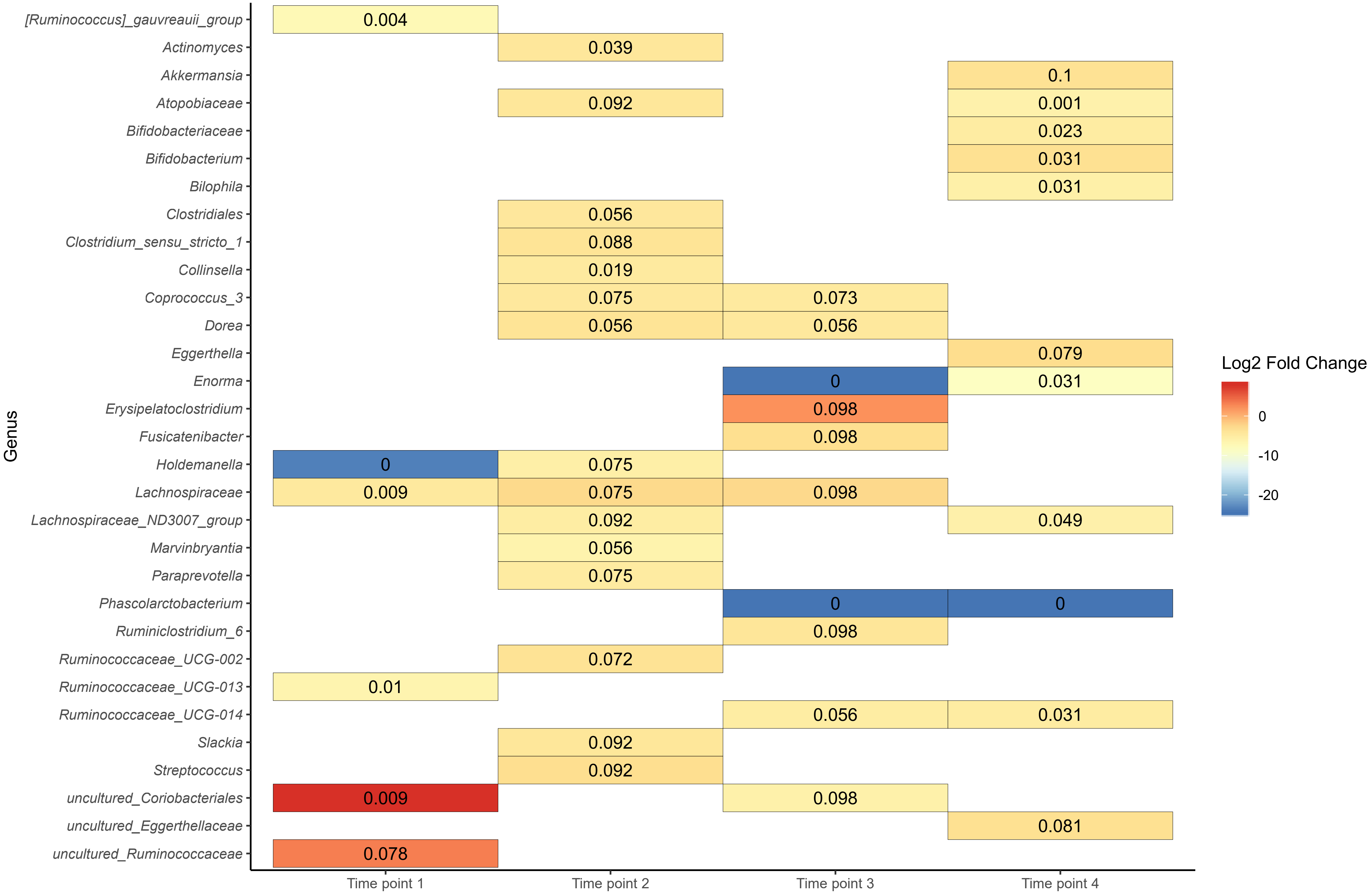

Fig S4: Heatmap showing differentially abundant genera between MDRO-positive samples and MDRO-negative samples. Log2FoldChanges changes with a positive number indicate genera that are more abundant in MDRO-positive samples, while log2FoldChanges with a negative number indicate genera which are more abundant in MDRO-negative samples. Adjusted p-values are displayed in the heatmap. A full overview can be found in Additional file 2: Table S1.

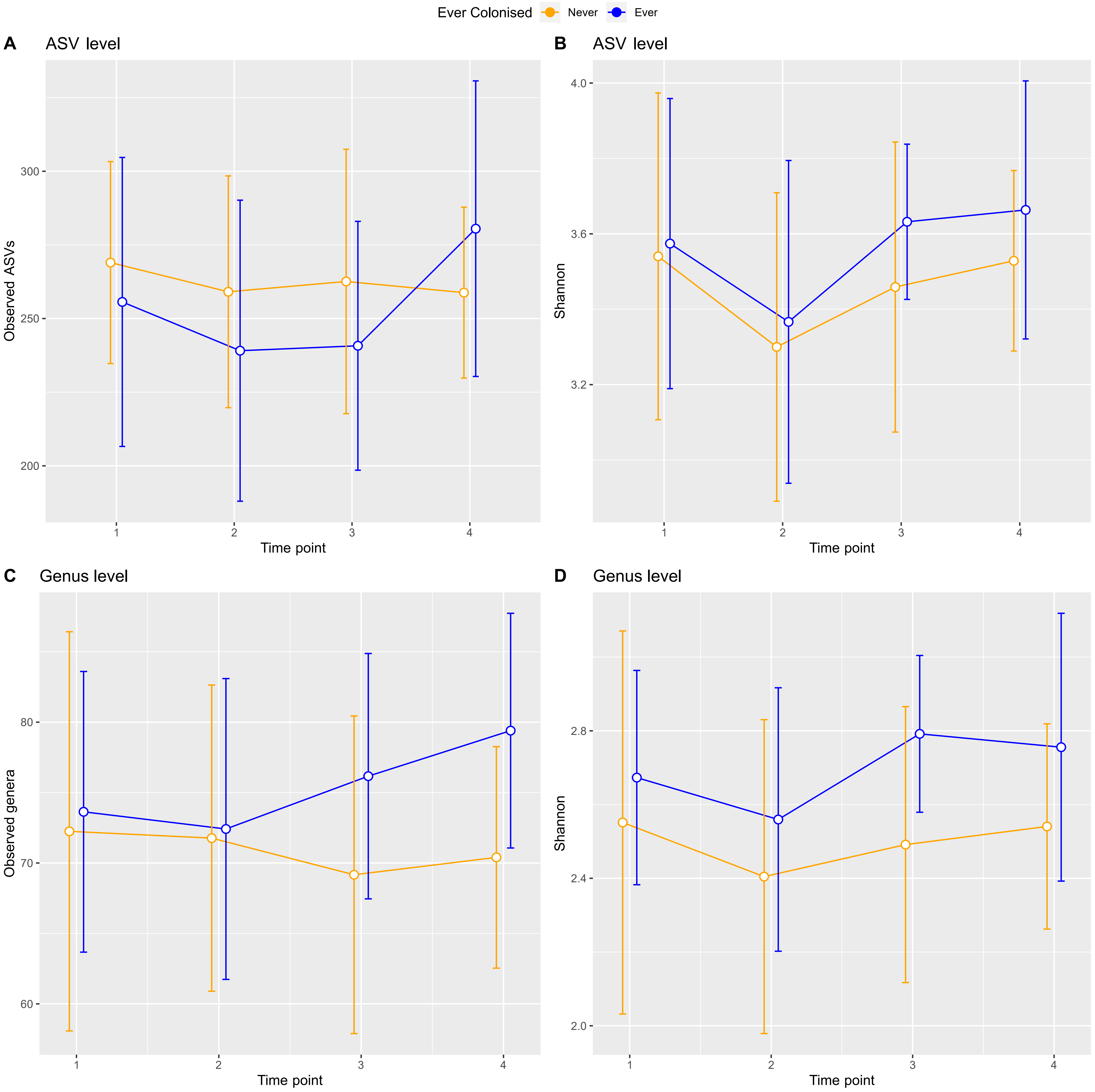

Figure S5: Alpha diversities at ASV (A + B) and genus level (C +D) for samples of twelve residents who were ever colonised versus fifteen residents who were never colonised. Linear mixed models were applied to investigate changes between the groups over time. Means and 95% CI of the standard error of the mean are shown.

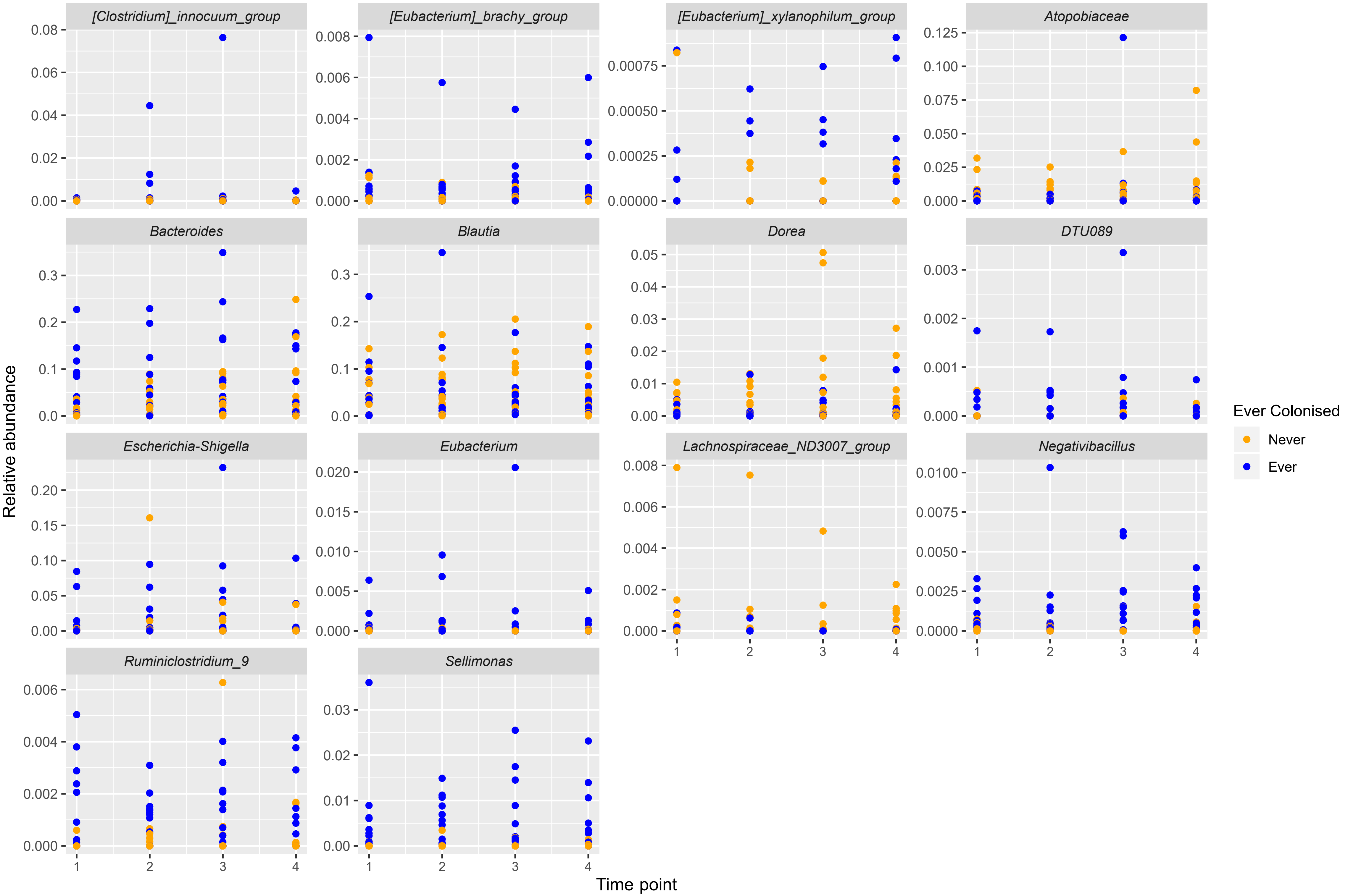

Figure S6: Relative abundances of differentially abundant genera identified by MetaLonDA, Each dot represents a single sample, and dots are coloured based on group (orange for n=12 residents ‘ever’ colonised, blue for n=15 residents ‘never’ colonised).
